# Cocaine Sensitization Increases Kyphosis and Modulates Neural Activity in Adult Nulliparous Rats

**DOI:** 10.3390/brainsci2040667

**Published:** 2012-11-27

**Authors:** Benjamin C. Nephew, Martha K. Caffrey, Ada C. Felix-Ortiz, Marcelo Febo

**Affiliations:** 1 Department of Biomedical Sciences, Tufts University Cummings School of Veterinary Medicine, North Grafton, MA 01536, USA; 2 Department of Psychology, Northeastern University, Boston, MA 02115, USA; Email: caffrey.m@husky.neu.edu (M.K.C.); felix-ortiz.a@husky.neu.edu (A.C.F.-O.); 3 Department of Psychiatry, University of Florida, Gainesville, FL 32610, USA; Email: febo@ufl.edu

**Keywords:** cocaine, sensitization, maternal behavior, kyphosis, crouching, nursing, hippocampus, thalamus, periaqueductal gray

## Abstract

Although data from both animals and humans suggests that adult cocaine use can have long term effects on behavior, it is unknown if prior cocaine use affects future maternal behavior in nulliparous females. In the current study, cocaine or saline was administered to adult female rats for 10 days, the animals were withdrawn from cocaine for 7 days, and the females were then exposed to donor pups to induce the expression of maternal behavior. Nulliparous females sensitized to cocaine were more likely to retrieve pups, spent more time caring for the pups, and were more likely to express full maternal behavior on day 8 of pup exposure. The fMRI data revealed significant effects of pup exposure in the hippocampal CA1 region, and effects of cocaine in the anterior thalamus and periaqueductal gray. Prior adult cocaine use may have lasting effects on offspring care, and this effect is not dependent on pup mediated effects or the endocrine changes of gestation and lactation. The present findings provide support for the hypothesis that maternal motivation to exhibit maternal behavior is enhanced by prior cocaine sensitization, possibly due to cross sensitization between cocaine and the natural reward of maternal behavior.

## 1. Introduction

It has been estimated that 15 million women in the US have used cocaine during adulthood, and this use often occurs during the reproductive years [1]. While it has been reported that recovering cocaine addicts display impaired maternal care [2,3], the mechanisms mediating these effects have not been explored in detail. Recovered addicts report that parenting is more stressful and they are more likely to rely on ineffective parenting techniques [4]. Recent studies suggest that long-term effects of cocaine can be modeled in rodents. Considering the importance of parenting on the physical and mental health of male and female offspring, it is possible that prior cocaine use has multi-generational effects on offspring mediated through parental behavior. The risk of negative effects of cocaine on offspring is not eliminated by abstinence. The objective of this study was to investigate the effects of adult cocaine exposure on the maternal behavior of nulliparous females caring for foster young. 

Cocaine exposure during gestation and/or lactation can significantly impair maternal behavior in rats, including disruptions of pup retrieval and crouching over the pups to nurse [5,6]. Acute cocaine treatment of lactating rats decreases maternal care of pups [7,8] and suppresses maternal aggression towards a novel male intruder [8]. In contrast to these studies of gestation or lactation, only a few previous investigations have examined the effects of prior use. Recent study using a novel model of prior chronic cocaine treatment in adult nulliparous rats concluded that adult cocaine exposure enhances subsequent primiparous pup retrieval [9] and ongoing maternal care and aggression [10]. These studies support the initial reporting of this phenomenon by Petruzzi *et al.* in 1997 [11]. It is postulated that there is cross-sensitization between cocaine exposure and pup retrieval, resulting in the increased display of maternal behavior. 

While studying the effects of prior cocaine use in lactating mothers will surely provide insight into the traditional parenting environment, many children in the U.S. receive a substantial amount of parenting from non-lactating females, such as relatives and daycare providers. In 2010, 16 million children received some form of regular child care from persons other than their parents [12], and 2.9 million children did not live with either parent [13]. The investigation of the effects of prior cocaine use on the maternal behavior of nulliparous virgins in this study may be relevant for millions of women and children. It is also a valuable model for determining if the effects of prior cocaine use on maternal behavior are dependent on the multitude of hormonal and neural changes that occur during gestation and lactation.

The current study was designed to expand on the previous literature reporting enhanced maternal care and aggression in lactating rats previously sensitized to cocaine to nulliparous rats induced to display maternal care through continuous exposure to pups. Nulliparous rats treated with cocaine as adults were tested for locomotor sensitization to the cocaine regimen, withdrawn from cocaine, and then tested for maternal care and aggression during 8 days of continuous exposure to young pups. It was hypothesized that prior chronic cocaine sensitization, a model of addiction, would enhance overall maternal behavior in nulliparous rats. The results provide insight into the expression of maternal behavior in nulliparous mothers with a history of cocaine use, and allow for the comparison of the effects of cocaine in nulliparous and primiparous females. Furthermore, the use of a maternal behavior model consisting of nulliparous females continuously exposed to young pups will test whether the increase in maternal behavior in rats previously treated with cocaine is dependent on the hormonal and/or neural changes associated with gestation and lactation. 

## 2. Results and Discussion

### 2.1. Sensitization to Cocaine

**Figure 1:**
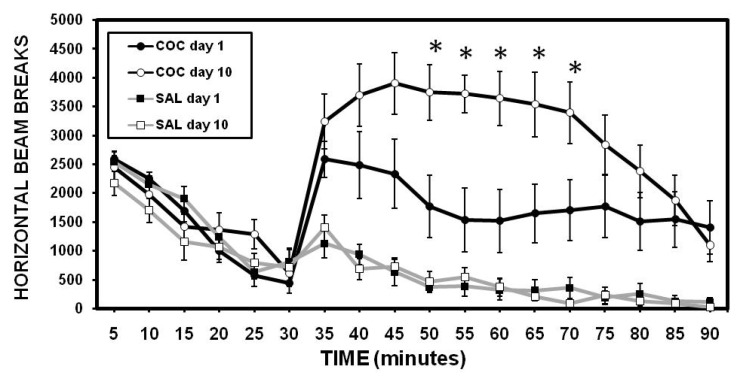
Mean ± SEM number of horizontal beam breaks in 5 min bins on day 1 and day 10 during 90 min activity trials. Saline (SAL *n* = 8) or 15 mg/kg cocaine (COC *n* = 9) injection were given IP at 30 min. * Indicates a significant difference between COC day 1 and COC day 10 (*p* < 0.05).

Locomotor activity was assessed for cocaine and saline treated adult females on days 1 and 10 of the 10 day treatment regimen. There were no significant differences between the four groups (saline and cocaine treated animals on days 1 and 10) during the initial 30 min pre-treatment period, and subsequent treatment comparisons used the 35 to 90 min time interval (12 five-minute bins) to test for activity level differences between saline and cocaine treated females (Figure 1). The locomotor response to IP cocaine was heightened on day 10 compared to day 1, an effect indicative of the development of behavioral sensitization to repeated cocaine treatment. Cocaine treated females exhibited increased horizontal movement (beam breaks, *F*_3,11_ = 146, *p* < 0.01, Figure 1) and total distance traveled (cm, *F*_3,11_ = 146, *p* < 0.01, data not shown) on day 10 of cocaine treatment compared to day 1. There were also significant effects of time (60 min period following treatment injection) on both horizontal movement (beam breaks, *F*_3,11_ = 5.4, *p* < 0.01, Figure 1) and total distance traveled (cm, *F*_3,11_ = 3.8, *p* < 0.01, data not shown).

### 2.2. Maternal Care

Maternal behavior was rarely observed on day 2 of pup exposure. Pup retrieval was only recorded in three animals during the day 8 maternal behavior test. This low level of retrieval was at least partially due to the lack of cage separators to prevent pups from crawling back to the nest (consistent with prior studies). All three retrieving females were cocaine treated (3/9 *vs.* 0/8, *p* = 0.08).

**Figure 2:**
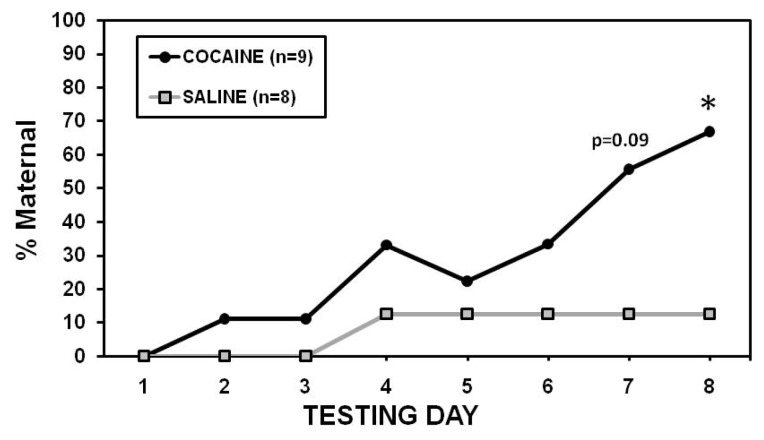
Percentage of saline and cocaine treated nulliparous rats that maternally responded to the presentation of foster pups over 8 days of continuous pup exposure. * Indicates a significant difference between saline and cocaine treated rats (*p* < 0.05).

**Figure 3:**
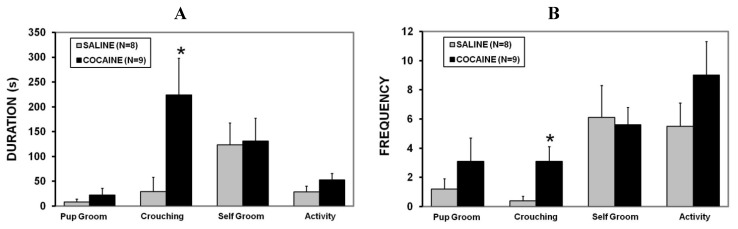
Mean ± SEM seconds (s) duration (**A**) and frequency (**B**) for four behaviors during a 15 min maternal care test on the 8th day of pup exposure in nulliparous rats previously treated with 10 consecutive days of saline or 15 mg/kg cocaine as adults. Samples sizes listed in parentheses. * Indicates a significant difference between saline and cocaine for an individual behavior (*p* < 0.05).

On day 8 of maternal testing, a greater proportion of cocaine treated nulliparous females displayed full maternal behavior (as defined as pup grooming and kyphosis) compared to saline treated controls (6/9 *vs.* 2/8, *p* = 0.04, Figure 2). There was also a trend for increased maternal responding in the cocaine group on day 7 (*p* = 0.09, Figure 2). Cocaine treated females spent more time crouching over the pups in a kyphotic posture (223.7 ± 74.1 *vs.* 29.0 ± 29.0 s, Figure 3A) and crouched more often (3.1 ± 1.0 *vs.* 00.4 ± 0.3, Figure 3B), during the 15 min maternal care observations on day 8. There were no effects of adult cocaine treatment on locomotor activity, self-grooming, or pup grooming (Figure 3).

### 2.3. Maternal Aggression

There were no effects of cocaine treatment on the aggressive responses to a novel male intruder. In general, the females displayed low levels of aggression. No maternal aggression was noted on day 2, and the few attacks observed on day 8 were preceded by long latencies in both saline and cocaine treated females. Although 7 out of 9 cocaine treated animals attacked the male intruder *vs.* 3 out of 8 saline treated females on day 8, there was no significant group difference (*p* = 0.11).

### 2.4. fMRI

**Figure 4:**
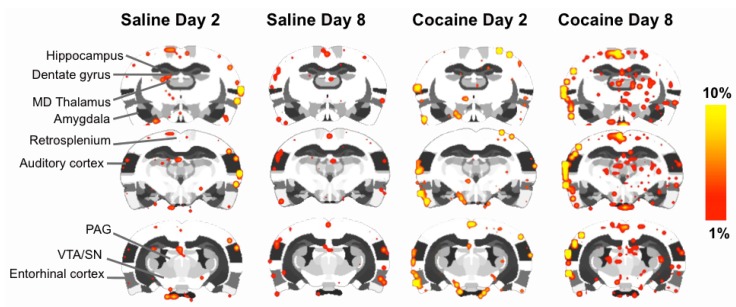
Composite activation maps showing areas of increased BOLD activation in rats presented with an empty presentation stage, stage with pups, and pups in the presence of a male intruder rat. Data are shown for control and cocaine treated rats on day 2 following initial exposure to pups and on day 8 after repeated sessions of exposure in their home cage. Color scale hue indicates percent change in signal intensity. Regions of interest are indicated to the left.

**Figure 5:**
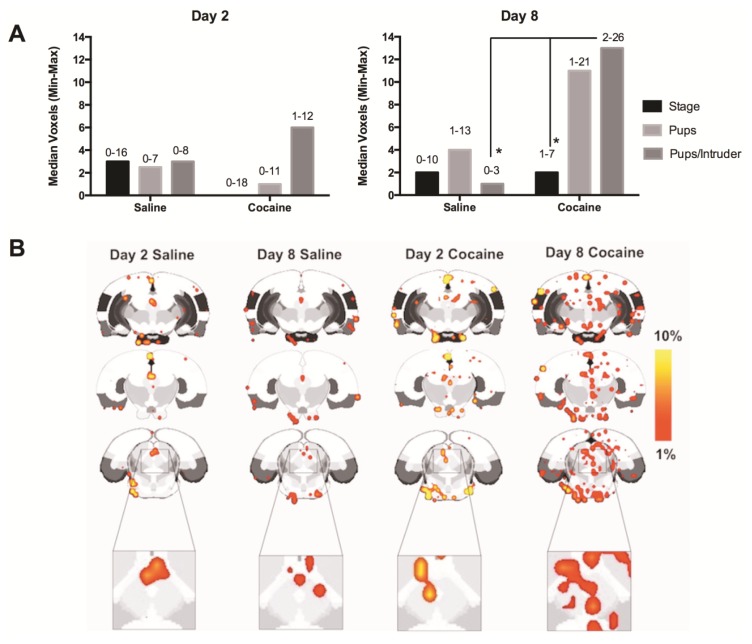
Median activated voxels in the PAG of saline and cocaine treated females presented with an empty stage, stage with pups, and pups with a male intruder rat on days 2 and 8 of pup sensitization (**A**), and composite activation maps showing areas of increased BOLD activation in rats presented with stage, stage with pups and pups and male intruder (**B**). Insets at the bottom highlight the area of the periaqueductal grey. Data are shown for control and cocaine treated rats on day 2 following initial exposure to pups and on day 8 after repeated sessions of exposure in their home cage. Color scale hue indicates percent change in signal intensity.

A total of 30 regions of interest were analyzed. However, particular focus was placed on ROI previously reported in primiparous animals using the same methods [14]. Both percent changes in BOLD and number of activated voxels were analyzed. However, as in our previous studies, we mostly observed changes in the volume of activation rather than the magnitude of BOLD signal change. A two-way analysis of variance with fMRI stimulus (stage, pups, pup/intruder) and cocaine treatment (saline *vs.* cocaine) as independent variables was used on day 2 and then on day 8 (Bonferroni’s multiple comparison posthoc test used with corrected alpha at 0.05). We did not observe any effect of drug or fMRI stimulus on day 2 for both percent changes in BOLD and number of voxels in any of the ROI tested. On day 8, we did observe an effect of fMRI stimulus presentation in the dorsal CA1 hippocampal area (Kruskall-Wallis ANOVA *F*_2,28_ = 3.5, *p* = 0.04) (Figure 4). Thus, both pups and pups/intruder increased BOLD signal distribution at greater levels than the stage alone. However, no effect of cocaine treatment was observed. Thus, the same response was observed in both saline and cocaine treated animals. An effect of cocaine treatment was observed, however, in both the anterior thalamic nuclei (Kruskall-Wallis ANOVA *F*_2,28_ = 4.3, *p* = 0.048) and the PAG (Kruskall-Wallis ANOVA *F*_2,28_ = 8.5, *p* = 0.0069). In the anterior thalamus, cocaine treated animals showed significantly greater activation than saline animals. The PAG showed a robust activation in response to intruder presentation (Figure 5). This appeared greatest in cocaine treated animals.

### 2.5. Discussion

Sensitization of adult female rats to cocaine decreased the latencies to initiate maternal care towards foster pups and specifically increased the display of kyphosis. The cocaine treated rats displayed a robust locomotor sensitization to cocaine, and these rats were more likely to respond maternally to foster pups. The fMRI data indicate that these behavioral responses were associated with altered neural responses in several nuclei involved in the control of maternal behavior. These effects were present two weeks following the cessation of cocaine sensitization. As in primiparous females, it is postulated that this increase in maternal behavior is due to cross-sensitization between cocaine and the natural reward of maternal behavior. Considering that millions of nulliparous females in the United States have used cocaine as adults, and that many of these women care for children through caring for family members, foster care, or working in the child care industry, the significant effects reported in the current study suggest that prior drug use may have long term effects on the adult maternal behavior of many women. 

The current data support and expand on the results of Febo and Ferris 2007 [9] and Nephew and Febo 2010 [10], which reported that cocaine prior to pregnancy decreased latencies for pup retrieval and increased the displays of maternal care and aggression. Although the current behavioral effects are not as robust as the documented changes in cocaine treated primiparous females, there are several potential explanations for this. Given that the majority of animals did not maternally respond until they were exposed to pups for 7–8 days, it is likely that the behavioral differences between the two groups would have been greater if the testing had been carried out for 10–12 days. It has been reported that nulliparous maternal care is not as robust as primiparous maternal care during the early stages of induction [15,16,17]. These studies have specifically noted that retrieval levels tend to be low in nulliparous models of maternal care. Furthermore, the logistics of the maternal care test made it difficult to record effects of prior cocaine on pup retrieval, which was documented in the prior reports. This was due to (1) a lack of cage separators to prevent pups from crawling to the next and (2) the inherently slow retrieval associated with nulliparous maternal care. The lack of cage separators was purposefully done to maintain consistency with the prior work from the Febo lab. It is likely that the differences between the two groups would have been greater if testing had been carried out for a longer period of time, and/or cage separators were used. The specificity of the effects of prior cocaine use is also consistent with the previous reports from primiparous females. The elevated maternal care was not associated with a general increase in locomotor activity. However, the noted specificity should be placed in the proper context. Potential anxiety or stress-mediated effects of prior cocaine exposure were not assessed in the current study, but it is possible that the effects of cocaine are mediated through a decrease in pup-associated fear or anxiety. It is possible that maternal care would not be increased in an environment with access to cocaine or cocaine related cues, as cocaine treated females may devote more time to investigating cocaine cues. Lastly, the current study documents an effect of cocaine on a reflexive maternal response, kyphosis, but not on behaviors more often associated with maternal motivation, such as retrieval and pup grooming [18,19]. However, these behaviors were increased by prior cocaine exposure in primiparous dams [10]. It is postulated that this lack of effect of cocaine on motivation associated maternal behaviors is due to the use of the nulliparous model, where maternal behavior is not as robust. Future studies will compare the motivation for both pups and cocaine in primiparous females previously treated with cocaine. 

Chronic cocaine treatment during gestation and lactation disrupt maternal retrieval and kyphotic nursing [5,6], but others have reported that these effects dissipate 24 h following cocaine injection [20]. However, while gestational cocaine disrupts maternal care, adult exposure results in decreased latencies and the increased expression of maternal care [10]. The current study also focused on the long-term effects of cocaine exposure, similar to the effects of amphetamine sensitization on sexual motivation in male rats [21], where prior amphetamine sensitization decreases male sexual behavior latencies. Fiorino and Phillips conclude that behavioral sensitization following repeated psychostimulant administration can cross-sensitize to a natural motivated behavior. After comparing these effects in males with our recent studies on cocaine and maternal behavior, it is postulated that prior cocaine exposure increases maternal behavior through a similar cross-sensitization mechanism. Pups are a powerful stimuli for inducing reward mediated behaviors in primiparous females [22,23,24], and the increase in maternal care in the cocaine treated nulliparous females suggests that the treated animals found the pups more rewarding than the saline treated animals. Future studies can test this hypothesis by measuring preference and intake of a saccharin solution *vs.* water. The divergent effects of adult and gestational exposure to cocaine may be due to the disruption of the neuroendocrine events of pregnancy. Maternal care is established during pregnancy, but in the adult exposure model, there is no overlap between cocaine treatment and the sensitization to pups. Other recent studies on maternal behavior provide insight on how adult cocaine sensitization may be affecting subsequent behavior.

Two likely neuroendocrine mediators for the effects of adult cocaine on maternal care are vasopressin (AVP) and oxytocin (OXT). AVP mediates the expression of maternal care [25,26] and is involved in the retention of the behavior in the absence of pups [27]. AVP has also been associated with the process of sensitization to cocaine [28,29]. The effects of adult cocaine exposure on subsequent maternal care may be mediated by increases in central AVP which occur during sensitization. Another possibility is that increases in amygdalar AVP during withdrawal from cocaine [30] may result in the subsequent increase in maternal care. Increased OXT activity in the hypothalamus may also be involved in cocaine induced increases in maternal care [11]. In support of this hypothesis, gestational cocaine suppresses maternal care and decreases OXT levels in the ventral tegmental area, hippocampus, and medial preoptic area [31]. Acute cocaine injections on lactation day 1 decrease OXT levels in the medial preoptic area [32], and this treatment is associated with decreased maternal care [8]. It is postulated that adult cocaine associated increases in maternal care are mediated by increases in central AVP and/or OXT activity.

One potential confound inherent in the earlier primiparous study of the long-term effects of cocaine on maternal care is the potential effects of prior cocaine on pup related stimuli, such as odors and vocalizations. Do pups from cocaine treated moms emit different stimuli (odors, vocalizations) compared to saline treated moms due to direct or indirect gestational effects? The current data suggest that differences in pup responses are not critical for prior cocaine induced increases in maternal care, as all the pups in the current study were raised by identically treated foster mothers. A second issue addressed with the use of the nulliparous sensitization model is the requirement for the hormonal changes of pregnancy, parturition, and lactation. OXT, AVP, prolactin, estradiol and progesterone all undergo significant fluctuations during gestation and/or lactation [33,34,35]. Nulliparous females sensitized to show maternal behavior do not go through the same changes. Adult cocaine exposure can affect maternal care in females independent of the endocrine changes associated with pregnancy and lactation.

The lack of significant aggressive responses in either group is likely to be due to the length of the pup sensitization period. Prior studies report that nulliparous females sensitized to express maternal care fail to express maternal aggression unless the sensitization period is extended for several days beyond the initial display of maternal care. It was hypothesized that endocrine changes associated with pseudopregnancy are required to induce maternal aggression in nulliparous females [36]. An extended maternal behavioral sensitization period in future studies may reveal cocaine induced increases in maternal aggression, as reported with adult cocaine exposure in primiparous females [10].

The fMRI results support and expand on previous neural activity studies of maternal care. A lack of specific BOLD responses to the distal pup presentation parallels the absence of Fos activity following distal stimulation from pups [37], but there were significant effects of time, cocaine treatment, and the novel male intruder presentation in several brain regions known to mediate maternal behavior. All three of the nuclei exhibiting significant changes in activated BOLD voxels, CA1, anterior thalamus, and PAG, have been implicated in earlier imaging studies of the central responses to the distal presentation of a novel male intruder [14,38] and previous research on maternal behavior.

Compared with nulliparous females, primiparous rats demonstrate enhanced reference or working memory [39,40,41], and this effect of parity may be mediated by dendritic remodeling in the CA1 and CA3 regions of the hippocampus [42,43]. Natural variations in rodent maternal care are associated with changes in glucocorticoid receptor mRNA levels [44] and innervation [45] in the hippocampus. In mice, double mothering enhances the performance of the offspring on hippocampal dependent tasks and increases dendritic length and spine density in the CA1 region [46]. Cocaine treatment during pregnancy in rats also increases dendritic spine density in the CA1, and it was concluded that pregnancy makes the brain more vulnerable to cocaine [47]. In the current study, it is hypothesized that maternal sensitization enhanced the innervation and/or dendritic structure of the CA1 region, which mediated the increases in neural activity in both the saline and cocaine treated females. In primiparous rats, the anterior thalamus responded specifically to the presentation of a novel male intruder, but not pups, in our earlier investigation [14]. It has also been implicated in the neural responses of rodents pups to maternal calls [48]. In humans, the thalamus is activated in response to the cry of a mother’s own infant in fMRI studies [49,50]. Cocaine treatment of nulliparous females in the present study sensitized the anterior thalamus, and this effect was associated with increased maternal care which may be a result of increased responsiveness to pup related stimuli. Lesions to the PAG cause a substantial reduction in kyphotic nursing [37,51], and cocaine treated females increased both kyphosis and neural activity in the PAG over time, which supports the initial lesion studies. Given the current intruder male imaging paradigm and the intruder specific voxel increase in the anterior thalamus PAG, it is interesting to note that the PAG has also been specifically linked to the initiation of maternal aggression [51]. Neuronal activity also increases in the PAG following the expression of maternal aggression [52]. It is hypothesized that the cocaine induced increases in PAG BOLD activity are evidence of the development of maternal aggression, but this is speculative due to the lack of aggressive behavioral data. As previously mentioned, an increase in maternal aggressive may have been observed if the pup sensitization period had been extended [36].

## 3. Experimental Section

### 3.1. Animals

Female Long-Evans rats (200–225 g) were purchased from Charles River Laboratories (Wilmington, MA, USA). Females were initially housed in pairs in a temperature and humidity controlled room and maintained on a 12L:12D light-dark cycle (lights off at 19:00 h). Home cages consisted of hanging plastic microisolater cages of standard dimensions with woodchip bedding. Water and Purina rat chow were provided *ad libitum*. Focal females used for behavioral analysis were singly housed along with the donor pups during maternal behavior sensitization. Donor females were singly housed following mating, and kept with their pups throughout the study. Rats were acquired and cared for in accordance with the guidelines published in the Guide for the Care and Use of Laboratory Animals (National Institutes of Health Publications No. 85-23, Revised 1985) and adhere to the National Institutes of Health and the American Association for Laboratory Animal Science guidelines. The Institutional Animal Care and Use Committee at Northeastern University approved the protocols used for this study. Sample sizes for behavioral analyses were 8 for the saline control treatment and 9 for the cocaine treatment. The procedural sequence was fMRI acclimation, cocaine sensitization, and maternal behavioral sensitization and testing and fMRI scanning.

### 3.2. Cocaine Treatment and Sensitization

Nulliparous adult females were injected intraperitoneally with either 0.1 mL of saline (*n* = 8), or a 15 mg/kg dose of cocaine in 0.1 mL saline (*n* = 9) daily (between 09:00 and 12:00) for 10 consecutive days. Animals were assessed for locomotor sensitization to cocaine between 09:00 and 12:00 on days 1 and 10 of the injection sequence. Female rats were placed in Automated Activity Cages (Columbus OH) and baseline locomotor activity recorded for 30 min. The cages are clear Plexiglas (Dimensions: 40 cm^2^) surrounded by a metal frame containing infrared photocell beams along its length and width (*X*-*Y* beams). Photocell interruptions along the *X*-*Y* axis register rat locomotor activity. The animal activity cages are interfaced with windows based software that allows the control of experiment start and end time independently for each subject. Following the 30 min baseline measurement, rats were given a saline or cocaine injection (15 mg/kg, intraperitoneal), and locomotor activity was then recorded for an additional 60 min. All activity data were grouped into 5 min bins for statistical analysis. Saline and cocaine treatments ended 7 days prior to the start of maternal behavior sensitization. 

### 3.3. Maternal Behavior Sensitization

On the first day of pup exposure, between 09:00 and 12:00 h, 3 foster pups, 3 to 10 days of age, that were recently fed (indicated by the presence of milk bands) were selected from lactating donor mothers. The test pups were allowed to remain with the focal females until the next day. The next day (09:00–12:00 h), after a 1-h waiting period, 3 recently fed pups obtained from lactating donor rats were placed in the test cages for maternal care testing. All saline and cocaine treated nulliparous females were presented with pups daily for 8 days. 

### 3.4. Behavioral Testing

Maternal care was assessed immediately following the introduction of 3 pups on each day of pup exposure. Testing consisted of the introduction of 3 fosters pups to the home cage and direct observation for 15 min. On days 2–8 of the pup exposure period, the pups from the previous day were removed for one hour prior to the introduction of new, fed pups for the maternal care testing. If the female grouped the pups together and crouched over them in a nursing posture, full maternal behavior was noted. Maternal behavior was confirmed through additional daily testing throughout the 8 day period. Females that initially expressed maternal care on day 8 received an additional test on day 9 to confirm that the behavior was consolidated.

Maternal care and maternal aggression were video recorded for 15 min on days 2 and 8 of pup sensitization for more comprehensive behavioral scoring and analysis. For maternal care testing, the frequency and duration of pup retrieval, pup grooming, kyphosis (crouching over pups with an arched back posture), nesting, self-grooming, and general locomotor activity were recorded using ODlog behavioral analysis software (Macropod Inc., Australia). The ODlog software records continuous data in 5 s bins, and also generates frequency and duration summaries for all behavioral measures over the 15 min observation period. 

Following the maternal care testing, the pups were removed and placed in a glass beaker adjacent to the water spout hole in the cage where the female had olfactory, auditory, and visual contact with them, and a novel male was placed in the cage for 15 min to test for maternal aggression. It is known that pup presence affects maternal aggression [53], and while pup removal was necessary to avoid attacks from the male, they were placed as close as possible to the dams to avoid attenuation of aggressive responses. Maternal aggression towards a novel male intruder included recording the latency to attack, and scoring the frequency and duration of attacks, as well as vigilance towards the intruder. Attacks consisted of bites, pummeling with forelimbs or hindlimbs, and pinning the male intruder to the floor of the cage. A single attack started upon contact between the male and female, and concluded when they separated. Vigilance describes the focused attention of a maternal female towards an intruder following the initial attack. Self-grooming and locomotor activity were also recorded using the ODlog software. 

### 3.5. Behavioral Statistics

The saline and cocaine treated group activity levels (cocaine sensitization) on days 1 and 10 of the injection sequence were compared with 2-way repeated measures analysis of variance (ANOVA, followed by Tukey’s *post hoc* tests) with treatment and time as factors. Fisher’s Exact Probability tests were used to compare the number of animals expressing full maternal behavior on each day of pup exposure. Individual ANOVAs were used to test for treatment differences in behavior on days 2 and 8 of maternal behavior sensitization. Data are presented as means ± SEM. Significant differences were denoted as *p* ≤ 0.05.

### 3.6. Acclimatization Procedures and Preparations for Imaging

All imaging experiments were done in fully awake, unanaesthetized primiparous dams. Anesthesia (2%–4% isoflurane) was used only during rat setup, preceding the acclimatization procedures and experiments. In order to minimize physiological and gross motion during MR scanning, all rats were acclimatized to a head restraining unit and MRI sounds upon arrival, one week prior to cocaine sensitization, as previously reported [54]. All fMRI scans were conducted on days 2 and 8 of pup sensitization between the hours of 13:00 and 17:00 following maternal behavior testing in the morning. Before MR scanning, dams were again anesthetized with 2%–4% isoflurane. Details of the setup procedure have been previously reported [22]. Briefly, a topical anesthetic of 5%–10% lidocaine cream was applied to the skin and soft tissue around the ear canals and over the bridge of the nose before the animal is placed inside a dual coil radiofrequency system under restraint [22]. This procedure took 5–6 min, after which gaseous anesthesia flow was turned off and the entire unit was placed through the bore of the magnet for imaging. After the entire unit was placed in the magnet, scanning preparations controlled by Paravision 4.0 typically took 10–15 min and thereafter the entire imaging session including 1 anatomical scan (*ca.* 6 min) and 3 functional scans (*ca.* 22 min total) lasted about 30 min. Thus the entire experiment per animal in an unanaesthetized state lasted 40–45 min.

### 3.7. Magnetic Resonance Imaging Scanning Parameters

Experiments were conducted in a 300 Mhz Bruker USR 7T/20 cm horizontal magnet (Bruker, Germany) equipped with a Paravision 4.0 console (Bruker, Billerica, MA, USA). Studies were performed with a multi-concentric dual-coil, small animal restrainer (Insight MRI, Worcester, MA, USA). Radiofrequency signals are sent and received with dual coil electronics built into the animal restrainer. Functional imaging was performed using a multisegmented T2 weighted fast spin echo pulse sequence with the following parameters: repetition time TR = 1562 ms, echo time TE = 7.5, effective echo time TEeff = 45 ms and an echo train length ETL = 16. Geometry was setup as follows: 12 slices, field of view of 28 mm, 1.0 mm thick slices with no gaps, data matrix of 642 for functional scans and 2562 for anatomical scans (Thus the in plane 2D pixel resolution was 438 μm^2^ for functional and 117 μm^2^ for anatomical scans). A full set of 12 coronal slices across the brain was collected at each effective repetition time and was completed every 6 s 24 ms.

### 3.8. Imaging BOLD Responses

Fourteen primiparous rats (6 saline and 8 cocaine) were imaged for the neural response to a control object (no pups), to object + pups (pups) and to nest intruder in the presence of pups (pups/intruder) on days 2 and 8 of pup sensitization. Each rat was imaged during 3 consecutive scanning sessions where an anatomical scan was first collected, immediately followed by a first fMRI scan of a control object (a custom-made delivery cradle alone without pups), a second fMRI scan of 4 pups (pups were presented on the delivery cradle), and a third and final fMRI scan of a male intruder in the presence of pups. For *in vivo* stimuli presentation, a custom made clear plastic cylindrical vivarium was used as previously reported [55]. The outer diameter of the vivarium tube is just under 20 cm, thus permitting it to remain stable inside the magnet bore in front of the dual coil imaging setup during functional and anatomical imaging. The front end of the vivarium has a non-magnetic copper wire mesh that permits dams to smell, visualize and hear pups or intruder males. The outer enclosure contains air holes and the inner environment of divided in top and bottom levels.

Maternal cage bedding is placed on the vivarium bottom floor to simulate the lactating rat’s home environment. For cradle alone and cradle + pups, 60 repetitions (6 min) were collected and stimulus presented a repetition number 30 (3 min). For intruder presentation, 100 repetitions (total 10 min) were collected and stimulus male presented in the vivarium at repetition 50 (5 min). During intruder male presentation, pups were placed in the lower compartment of the vivarium along with their home cage bedding to protect them from any harm during scanning. 

### 3.9. fMRI Statistical Analysis

Full details for the MRI data analysis using in house software has been previously reported [22] and are available as online supporting materials Animals showing an average displacement exceeding 25% of the total in plane (*X*-*Y*) pixel resolution (>109 μm out of 437 μm) or slice (*Z*) direction (>250 μm out of 1000 μm slice thickness) were excluded (*n* = 3). This a priori cutoff criterion was pre-established by stimulated studies showing false positive BOLD activation with movements corresponding to 6/10 of a single voxel [56]. Also, scans with linear baseline drifts over 0.5% were corrected using in house software [56]. Nine out of 14 studies survived the preprocessing exclusion criteria and were included in the study. ROI-based statistical analysis was done using Medical Image Visualization and Analysis (MIVA) software [22]. Each subject was registered to a fully segmented electronic rat brain atlas [57,58]. Statistical *t*-tests are performed on each subject within the original coordinate system. The baseline period used was 15 repetitions immediately preceding stimulus (object, pup, or intruder) presentation and the stimulation window was 15 repetitions. Statistical *t*-tests used a 95% confidence level, two-tailed distribution, and heteroscedastic variance assumptions. In order to provide a conservative estimate of significance, a false-positive detection-controlling algorithm is introduced into the analysis. This ensures that the false-positive detection rate is below our confidence level of 5% [22]. Statistically significant pixels were assigned their percentage change values (stimulus mean minus control mean) and all. Activated voxel numbers were exported to SPSS for statistical comparisons between groups. Unless otherwise noted in the results section, the number of voxels per region of interest and their corresponding average percent change values were statistically evaluated between groups using a multi-factorial analysis of variance (*p* < 0.05) with stimulus (no pups, pups, pups/intruder) as independent variables. A Bonferroni multiple comparison test was used for posthoc analysis. For statistical comparisons between intruder presentation, without and with pups, we used a single factor analysis of variance. Significance was denoted at *p* < 0.05 (two-tailed). Data were analyzed for both percent changes in BOLD signal intensity and volume of activation for both positive and negative BOLD responses.

## 4. Conclusions

In conclusion, the current data indicate that adult cocaine exposure increases the expression of subsequent pup-induced reflexive maternal care, and this behavioral effect is associated with elevated neural activity in regions known to mediate maternal behavior. The present findings support the hypothesis that the motivation of nulliparous female rats to exhibit maternal behavior is enhanced by cocaine exposure, possibly due to cross sensitization between cocaine and the natural reward of maternal behavior. This effect of cocaine on nulliparous maternal care may impact millions of foster mothers and women in the childcare industry who are not biological mothers but display maternal care towards the offspring of others.
